# Online Isotope Analysis of Sulfur in Proteins via Capillary Electrophoresis Coupled With Multicollector ICP‐MS (CE/MC‐ICP‐MS): A Proof of Concept Study

**DOI:** 10.1002/elps.202400128

**Published:** 2024-09-30

**Authors:** Dariya Tukhmetova, Nicole Langhammer, Jochen Vogl, Björn Meermann

**Affiliations:** ^1^ Division 1.1—Inorganic Trace Analysis Federal Institute for Materials Research and Testing (BAM) Berlin Germany

**Keywords:** CE/MC‐ICP‐MS, isotope analysis, sulfur

## Abstract

Isotope ratio analysis of sulfur in biological samples using inductively coupled plasma‐mass spectrometry (ICP‐MS) has gained significant interest for applications in quantitative proteomics. Advancements like coupling separation techniques with multicollector ICP‐MS (MC‐ICP‐MS) enhance the throughput of species‐specific sulfur isotope ratio measurements, fostering new avenues for studying sulfur metabolism in complex biological matrices. This proof‐of‐concept study investigates the feasibility of online CE/MC‐ICP‐MS for directly analyzing sulfur isotope ratios in proteins (albumin). Leveraging our previous work on the applicability of CE/ICP‐MS for quantifying sulfur‐containing biological molecules, we explore its potential for sulfur isotope analysis. Our results demonstrate that direct analysis of sulfur isotopes in albumin protein using online capillary electrophoresis MC‐ICP‐MS (CE/MC‐ICP‐MS) eliminates the need for laborious pretreatment steps, while yielding isotope ratios comparable to the reference values. Although initial precision can be improved through further system optimization and protein injection techniques, this approach paves the way for future analysis of mixtures of various biological compounds in, for example, clinical diagnosis studies.

**Abbreviations**
IAEA: International Atomic Energy Agency
IUPAC: International Union of Pure and Applied Chemistry
MC‐ICP‐MS: multicollector ICP‐MS
NIST: National Institute of Standards & Technology
PAI: peak integration method
s: standard deviation
S1: isotope reference material IAEA‐S‐1
S2: isotope reference material IAEA‐S‐2
S3: isotope reference material IAEA‐S‐3
SMIL: successive multiple ionic polymer layers
SRM: standard reference material
U: expanded uncertainty
VCDT: Vienna Cañon Diablo meteorite

## 1 Introduction

Although traditionally, isotopic analysis of mineral elements has been associated with geo‐ and cosmochemistry, as well as environmental sciences, there is a growing recognition of its relevance in life sciences. Recent advancements highlight its significance in understanding disease processes and guiding clinical research. Studies examining stable isotopes of essential mineral elements like copper, zinc, sulfur, calcium, iron, and others reveal nuanced variations linked to diseases such as cancer, liver cirrhosis, and metabolic disorders [1, 2, 3].

Studying sulfur isotopes in biological systems is important as sulfur is involved in essential processes such as amino acid synthesis, protein formation, and cellular respiration. During these metabolic activities, sulfur isotopes can undergo fractionation as they are assimilated into biomolecules. Unlike metal ions bound in coordination complexes, covalently bound sulfur is less susceptible to changes during sample processing and analysis, ensuring the robustness of results. By analyzing the delta value of sulfur (*δ* ^34^S) in tissues, fluids, or organisms, researchers can trace the sources of sulfur, infer metabolic pathways, and investigate physiological processes and pathological conditions [4, 5, 6].

Recent years have witnessed a development in isotope analysis of sulfur utilizing inductively coupled plasma‐mass spectrometry (ICP‐MS) for quantitative proteomics [7]. Emerging trends include the adoption of multicollector ICP‐MS (MC‐ICP‐MS) instruments for precise sulfur isotope ratio measurements and the utilization of sulfur stable isotopes in metabolism studies, promising new advances in this field. Biological samples often have diverse matrices and sulfur contents, requiring optimized methods for sample digestion, purification, and extraction of sulfur compounds. By online coupling ICP‐MS with chromatographic or electrokinetic techniques, hyphenated methods allow for improved throughput in detecting sulfur isotopes. Such separation techniques selectively isolate sulfur‐containing compounds from biological samples/matrices, minimize interferences and matrix effects, and, consequently, discriminate among different sulfur species and isotopic signatures [8].

Recent research has shown promise for analyzing sulfur isotopes (both ^3^ ^2^S and ^3^⁴S) in different materials using hyphenated methods. One study published by Kümmel et al. [9] presented a new method using gas chromatography coupled with MC‐ICP‐MS (GC/MC‐ICP‐MS) to analyze both *δ* ^33^S and *δ* ^34^S isotopes simultaneously in organic compounds. The method offers high precision and can be used at lower resolution settings for increased sensitivity, making it valuable for various applications, including the analysis of industrially produced organic compounds. Another study also used GC/MC‐ICP‐MS [10] to separate organic sulfur compounds from H_2_S before analysis, achieving high precision (better than 0.3%) and revealing potential variations in sulfur isotope ratios within individual compounds. Fassbender et al. [11] investigated the use of CE/MC‐ICP‐MS for analyzing sulfur isotopes in environmental samples, that is, river water samples from various locations. This research showed that CE/MC‐ICP‐MS could be just as precise as traditional offline methods, while deploying a special injection‐bracketing protocol and assessing various data processing options for transient signals. However, despite these advancements of hyphenated techniques used for species‐specific isotope analysis of sulfur in industrial organic and environmental samples, there is a lack of research applying these techniques to analyze sulfur‐containing (bio‐)molecules in life sciences.

As a proof‐of‐concept study, our goal is to show the possibility of using an online CE/MC‐ICP‐MS method for species‐specific isotope analysis of sulfur in complex biological compounds such as proteins (sulfur in albumin). Earlier, we have shown the feasibility of online CE/ICP‐MS for the reliable quantification of various sulfur‐containing molecules via an online isotope dilution method [12]. This article discusses further applications of such an online setup for isotope studies.

## 2 Materials and Methods

### 2.1 Chemicals

Purified water was from Milli‐Q water purification system (Merck Millipore, France). Concentrated trace metal analysis‐grade nitric acid (HNO_3_) and hydrochloric acid (HCl) were from Primar Plus, Thermo Scientific, Germany. Formic acid (98%–100% Suprapur grade from Merck KGaA, Germany) was used to prepare the background electrolyte (BGE) for CE. Ethanol (≥99.8%, Honeywell, USA) was added to the CE sheath liquid (5% (*v/v*)) to improve the aerosol formation via the CE interface nebulizer. For successive multiple ionic polymer layers (SMIL) coating [11] of the CE capillary, we used hexadimethrine bromide (polybrene) with ≥95% purity and dextran sulfate sodium salt with 40 000 g mol^−1^ from Merck KGaA, Germany. Isotope reference materials IAEA‐S‐1 (Ag_2_S; *δ* ^34^S = −0.30%) denoted as S1, IAEA‐S‐2 (Ag_2_S; *δ* ^34^S = 22.62%) denoted as S2 and IAEA‐S‐3 (Ag_2_S; *δ* ^34^S = −32.49%) denoted as S3 were from the International Atomic Energy Agency (IAEA) (Vienna, Austria). Sodium standard (prepared from 1000 mg L^−1^ NaNO_3_, TraceCERT grade) was from Merck KGaA, Germany. Bovine serum albumin (BSA), further referred to as albumin, 7% solution 927f standard reference material (SRM) was from the National Institute of Standards & Technology (NIST), USA. The amino acid sequence of albumin is known and contains 1.88% sulfur (*w/w*) as a constituent of methionine and cysteine amino acids [13].

### 2.2 Procedures

The fused silica capillary was SMIL coated according to Faßbender et al. [11], consisting of two layers of polybrene separated by a layer of dextran sulfate. Capillary electrophoresis benefits from SMIL coatings in several ways: reduced adsorption, improved separation efficiency and resolution of protein separation, and enhanced reproducibility of repeated measurements [14].

S1, S2, and S3 standards were digested following the procedure developed by Craddock et al. [15], including the addition of acids (concentrated HNO_3_ and HCl) to convert the sulfide to sulfate form, and evaporation to dryness at 70°C using a hot plate. However, the complete digestion of albumin was done using a high‐pressure washer (HPA‐S, Anton Paar GmbH, Austria) with 70‐mL quartz vessels. A volume of 0.3 mL NIST 927f solution was mixed with 1.2 mL concentrated nitric acid. The digestion program started with a temperature rise until 150°C during 30 min, and then the temperature was further increased to 320°C during 60 min and kept constant for 150 min at 100 bar and cooled down to room temperature for 60 min. Next, the digested protein sample was transferred to PFA beakers and dried at 70°C on a hot plate.

The dry residue samples obtained both from Ag_2_S digestion and protein digestion were redissolved in (i) Milli‐Q water to an S concentration of 1 g L^−1^ to be analyzed with CE/MC‐ICP‐MS and (ii) in 0.03 mol L^−1^ HNO_3_ to an S concentration of 5 mg L^−1^ for further purification. The purification was performed using an anion exchange resin (AG1X8; Cl form; 200–400 mesh; BIORAD Labs, Germany) following the procedure developed by Das et al. [16]. The solutions for MC‐ICP‐MS analysis were diluted to a final S concentration of approximately 1 mg L^−1^ in 0.3 mol L^−1^ HNO_3_ with an addition of 3 mg L^−1^ of Na to improve the transmission of S through the membrane desolvation system (Apex Ω membrane desolvation system [Elemental Scientific, USA]) [17].

### 2.3 Instrumentation

CE/MC‐ICP‐MS coupling was established using a MiraMist CE nebulizer (Burgener Research Inc., Canada). The sheath liquid was delivered at a constant flow rate using a 10 mL Hamilton glass syringe (Hamilton Company, USA) secured onto a syringe pump (Harvard Apparatus, USA). Detailed operating conditions are shown in Table 1.

**TABLE 1:** Operating conditions of the CE and multicollector ICP‐MS (MC‐ICP‐MS) system.

Parameter	Value
ICP‐MS Instrument	Neptune multicollector ICP‐MS (Thermo Scientific, Germany)
Desolvation system	Apex Ω membrane desolvation system (Elemental Scientific, USA)
Cones	Ni “X” Skimmer cone, Ni Jet Sampler cone (Thermo Scientific, Germany)
Acquisition	^32^S (L3), ^33^S (C), and ^34^S (H3), with 0.262 s integration time, at medium resolution on the lower mass shoulder
Gas flow	0.37 L min^−1^ sample gas (make‐up gas), 0.9 L min^−1^ auxiliary gas
CE instrument	Agilent CE 7100 (Agilent Technologies, Germany)
Capillary	75 µm i.d., 100 cm long fused silica with SMIL coating [11]
Background electrolyte	0.5 mol L^−1^ formic acid, pH ≈ 2
CE injection	Hydrodynamically, sulfate standard at 100 mbar for 50 s, albumin standard at 100 mbar for 20 s
CE run	−20 kV (35–37 µA) with applied internal pressure (20 mbar for sulfate standard, 50 mbar for albumin standard) at 22°C
CE post‐run	Flush with BGE for 90 s, wait 30 s
Sample	Single‐component solutions with 100 mg L^−1^ sulfur diluted in Milli‐Q water
Nebulizer	Burgener MiraMist CE
Gas pressure	Ar at 5.2 bar (75 psi)
Spray chamber	8 mL Quartz, drainless with make‐up gas
Sheath liquid	0.01 mol L^−1^ HNO_3_ with 5% (v/v) ethanol
Sheath liquid delivery	At 7 µL min^−1^ flow with syringe pump

Abbreviations: BGE, background electrolyte; ICP‐MS, inductively coupled plasma‐mass spectrometry; SMIL, successive multiple ionic polymer layers.

### 2.4 Data Processing

Background correction and delta calculation on the continuous signal were automatically carried out by the MC‐ICP‐MS instrument software. Instrumental conditions were optimized using the S2 and S3 standards. Processing of the transient signal generated with online CE/MC‐ICP‐MS was accomplished using the dedicated in‐house built tool IsoCor [18]. The IsoCor has an option to include normalization of delta values according to Equation (1) using a scaling coefficient *coef*. For the isotope analysis of sulfur, we normalized the delta values relative to Vienna Cañon Diablo meteorite (VCDT) scale using the S1 standard as recommended by IUPAC [19]. Considering that $\delta_{S1/VCDT}^{34/32}S=-0.0003$, the *coef* from Equation (1) was calculated as 0.9997 according to eqn. 9 & 10 in [18] to calculate $\delta_{smp/VCDT}^{34/32}S$ according to Equation (2). The peak integration method (PAI) was applied for isotope ratio calculation, as it proved to compensate for isotope ratio drifts [11]:

(1) $$\delta_{smp/std}^{i/j}=\left(\frac{R_{smp}^{i/j}}{R_{std}^{i/j}}\right)\times coef-1$$

(2) $$\delta_{smp/VCDT}^{34/32}S=\left(\frac{R_{smp}^{34/32}}{R_{S1}^{34/32}}\right)\;\times \left(\delta_{S1/VCDT}^{34/32}S+1\right)-1$$

Error propagation of the delta results of sulfur isotopes in BSA was conducted by estimating uncertainty contributors based on repeated measurements (to cover all contributions related with the measurement) using the example Excel worksheet by Vogl et al. [20] and denoted as *u_m_*. Then, in order to account for the uncertainty contribution related to the sample preparation, we included the difference between the measured (from CE/MC‐ICP‐MS) and the reference results (from MC‐ICP‐MS) to obtain a total combined uncertainty *u_c,t_* using Equation (3). A coverage factor *k* = 2 (95% confidence) was used to calculate the expanded uncertainty *U* = *u*_c,t_ × 2. The calculations in the Excel worksheet were based on the principles described in the Guide to the Expression of Uncertainty in Measurement (GUM), as published by the members of the Joint Committee for Guides in Metrology [21]:

(3) $$u_{c,t}=\sqrt{u_{m}^{2}+{\left(\frac{\delta^{34/32}S_{reference}-\delta^{34/32}S_{measured}}{2}\right)}^{2}}$$

## 3 Results and Discussion

**FIGURE 1:**
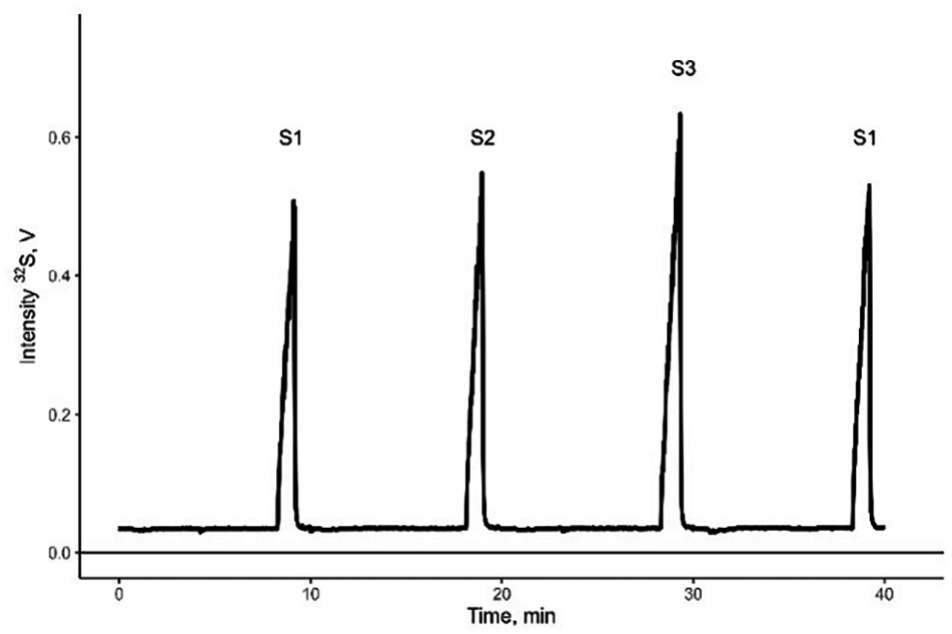
Electropherogram of sulfate standards (S1, S2, and S3). Each standard was injected separately and combined into one electropherogram during data processing. S1, isotope reference material IAEA‐S‐1; S2, isotope reference material IAEA‐S‐2; S3, isotope reference material IAEA‐S‐3.

A standard‐sample‐bracketing approach is commonly used for correcting instrumental mass bias in isotope analysis. However, adapting this approach to online hyphenated techniques, where samples are continuously introduced, presents a unique challenge. To address this, researchers have developed various “tricks” such as a multiple‐injection protocol for CE/MC‐ICP‐MS in which standard and samples are injected consequently and measured as one run [11]; injecting a standard several times at the start and end of each single run [9, 10]; or injecting standard and samples consequently as separate runs [22] for GC/MC‐ICP‐MS. We used the second approach (consequent injection of standard and sample as single runs) to minimize the total sample measurement time because one standard can be used as a bracketing standard for two samples. During data processing for the isotope ratio calculations, the separate runs of two standards and one sample were combined into one file and further processed with the IsoCor tool. To check the suitability of the system for the online analysis of ^32^S and ^34^S isotopes, we separately injected standards S1, S2, and S3 (Figure 1), where S1 was a bracketing standard for samples S2 and S3. The assigned reference values were compared with the measured values (Table 2) calculated with Equation (1). Two times standard deviation (2s) of six repeated measurements via CE/MC‐ICP‐MS were 16 and 22 times higher for S2 and S3 samples compared to the expanded uncertainties (*U*) documented in the IAEA reference sheet [23]. Such big discrepancy can be explained by the highly precise IRMS measurements conducted for the isotope reference materials, as well as an inherited lower precision of online measurements compared to traditional steady‐state offline MC‐ICP‐MS measurements.

**TABLE 2:** Comparison of *δ* ^34^S values for standards isotope reference material IAEA‐S‐2 (S2) and isotope reference material IAEA‐S‐3 (S3) measured by multicollector ICP‐MS (MC‐ICP‐MS) and CE/MC‐ICP‐MS, with the corresponding certified values.

	Assigned value [23] *δ* ^34^ *S_VCDT_* ± *U* (*k* = 2), %	MC‐ICP‐MS *δ* ^34^ *S_VCDT_* ± 2s, %	CE/MC‐ICP‐MS *δ* ^34^ *S_VCDT_* ± 2s, %	CE/MC‐ICP‐MS [11] *δ* ^34^ *S_VCDT_* ± 2s, %
S2 (IAEA‐S‐2)	22.62 ± 0.16	23.02 ± 0.08 (*n* = 6)	22.42 ± 2.59 (*n* = 6)	21.24 ± 0.23 (*n* = 3)
S3 (IAEA‐S‐3)	−32.49 ± 0.16	n.a.	−33.13 ± 3.57 (*n* = 6)	−32.20 ± 1.90 (*n* = 3)
BSA (NIST 927f)	n.a.	2.45 ± 0.07 (*n* = 3)	3.59 ± 2.24 (*n* = 6)	N.A.

Abbreviations: BSA, bovine serum albumin; IAEA, International Atomic Energy Agency; NIST, National Institute of Standards & Technology; n.a., not available; VCDT, Vienna Cañon Diablo meteorite.

Fassbender et al. [11] reported that with a similar setup, it is possible to measure *δ* ^34^S_VCDT_ with a better precision than is reported in this article. This can be explained by the sensitivity of the instrumental setup: 4.5 V [11] versus 0.6 V (Figure 1) corresponding to 5 and 40 ng sulfur injected, respectively. Considering the similar CE parameters used in both studies (75 µm i.d., 100 cm long fused silica capillary with SMIL coating connected to MC‐ICP‐MS using a MiraMist CE nebulizer with a similar sheath liquid flow rate), lower sensitivity of our system is most likely linked to inferior tuning of the ICP‐MS part. Impact of oxygen interference ^16^O_2_ when measuring ^32^S is well pronounced at medium resolution settings in MC‐ICP‐MS; thus, correct tuning of the system is critical to obtain optimal intensity and a stable signal for ^32^S. Furthermore, the sample/matrix analyzed within our current study is much more complex (proteins) than within our previous study on surface waters. Overall, results from Table 2 show that the accuracy is sufficient for our current needs, and we can proceed with the analysis of more complex samples.

**FIGURE 2:**
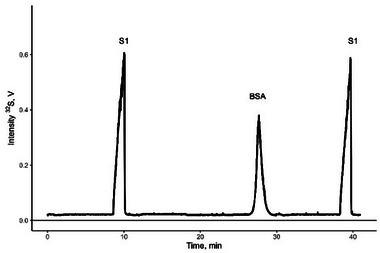
Electropherogram of sulfate (S1) and an albumin (BSA, NIST SRM 927f) standard. Each standard was injected separately and combined into one electropherogram during data processing. BSA, bovine serum albumin; NIST, National Institute of Standards & Technology; S1, isotope reference material IAEA‐S‐1; SRM, standard reference material.

To analyze isotope ratios of sulfur in organic molecules, we chose a pure albumin standard with its certified concentration and known amino acid sequence [13] to prepare a standard solution with a known concentration of sulfur. Higher internal pressure (Table 1) was applied when running albumin to obtain relatively sharp albumin peaks (Figure 2). Results of *δ* ^34^S_VCDT_ in albumin were comparable to the ones in S2 and S3 in terms of 2s precision when measured with our CE/MC‐ICP‐MS system (Table 2).

The error propagation calculations related to delta values of sulfur in S2 and S3 resulted in an expanded uncertainty of *U* = 1.01%, *k* = 2; in BSA, the expanded uncertainty was *U* = 1.46%, *k* = 2. The online system demonstrated comparable precision in measuring delta values in both S2, S3, and in BSA samples, as evidenced by the similar expanded uncertainties. However, the slightly higher uncertainty observed for the measurement of *δ* ^34^S in BSA suggests an influence of the type of sample contributing to the uncertainty. When comparing the expanded uncertainty (1.46%) with the twofold standard deviation 2s (2.24%) for *δ* ^34^S in BSA, the lower value of the expanded uncertainty can be attributed to the correlation between standard and sample, which reduces the uncertainty and is not considered or included in a standard deviation.

The empirical correlation coefficient between the sample and standard isotope ratios was determined to be 0.6, indicating a moderate positive relationship. Although this suggests that the standard‐sample bracketing approach is partially effective in correcting for instrumental drift, it also implies the presence of additional factors influencing the isotope ratios. Given the complexity of the CE/MC‐ICP‐MS system, several factors could contribute to the less‐than‐perfect correlation: Protein interactions within the capillary can introduce variability; fluctuations in nebulization efficiency; long‐term drifts in mass bias and short‐term fluctuations in plasma conditions. Some suggestions for improvement: optimizing the CE separation parameters to make run times shorter (BGE composition, pH, capillary length and inner diameter, etc.); testing other types of CE capillary coating to minimize the interaction of proteins with the CE capillary wall; and potentially employing large volume sample stacking approaches within the CE capillary to increase the amount of injected protein; improving the tuning of the MC‐ICP‐MS system, and use of Faraday cups with higher amplification.

## 4 Concluding Remarks

Our proof‐of‐concept study demonstrates the potential of directly analyzing sulfur isotopes in complex biological molecules like albumin protein using the online CE/MC‐ICP‐MS setup. This eliminates the need for time‐consuming sample pretreatment steps such as extraction or digestion when analyzing biological samples. Although the sulfur isotope ratios obtained with the online system were comparable to reference values from offline measurements, the precision could be improved. Given the complexity of the CE/MC‐ICP‐MS system, several factors likely contribute to this, including optimization of the CE parameters, the use of Faraday cups with higher amplification, improved tuning of the system, and potentially employing large volume sample stacking techniques within the CE capillary to increase the amount of injected protein.

The first results of sulfur isotope ratios upon albumin protein analysis promise future applications with mixtures of various biological compounds. Furthermore, the capability of the method could be further extended for analyzing environmental, pharmaceutical, and clinical samples for diagnosis.

## Conflicts of Interest

The authors declare no conflicts of interest.

## Data Availability

The data that support the findings of this study are available from the corresponding author upon request.
